# Crop-Zone Weed Mycobiomes of the South-Western Australian Grain Belt

**DOI:** 10.3389/fmicb.2020.581592

**Published:** 2020-11-24

**Authors:** Pippa J. Michael, Darcy Jones, Nicole White, James K. Hane, Michael Bunce, Mark Gibberd

**Affiliations:** ^1^Centre for Crop and Disease Management, School of Molecular and Life Sciences, Curtin University, Perth, WA, Australia; ^2^TRENDLab, School of Molecular and Life Sciences, Curtin University, Perth, WA, Australia

**Keywords:** mycobiome, phyllosphere, fungi, plant pathogen, weeds

## Abstract

In the absence of a primary crop host, secondary plant hosts may act as a reservoir for fungal plant pathogens of agricultural crops. Secondary hosts may potentially harbor heteroecious biotrophs (e.g., the stripe rust fungus *Puccinia striiformis*) or other pathogens with broad host ranges. Agricultural grain production tends toward monoculture or a limited number of crop hosts over large regions, and local weeds are a major source of potential secondary hosts. In this study, the fungal phyllospheres of 12 weed species common in the agricultural regions of Western Australia (WA) were compared through high-throughput DNA sequencing. Amplicons of D2 and ITS were sequenced on an Illumina MiSeq system using previously published primers and BLAST outputs analyzed using MEGAN. A heatmap of cumulative presence–absence for fungal taxa was generated, and variance patterns were investigated using principal components analysis (PCA) and canonical correspondence analysis (CCA). We observed the presence of several major international crop pathogens, including basidiomycete rusts of the *Puccinia* spp., and ascomycete phytopathogens of the *Leptosphaeria* and *Pyrenophora* genera. Unrelated to crop production, several endemic pathogen species including those infecting Eucalyptus trees were also observed, which was consistent with local native flora. We also observed that differences in latitude or climate zones appeared to influence the geographic distributions of plant pathogenic species more than the presence of compatible host species, with the exception of Brassicaceae host family. There was an increased proportion of necrotrophic Ascomycete species in warmer and drier regions of central WA, compared to an increased proportion of biotrophic Basidiomycete species in cooler and wetter regions in southern WA.

## Introduction

In the absence of a primary crop host, secondary plant hosts may act as a reservoir for fungal plant pathogens that cause economically significant crop diseases. In a crop monoculture environment, readily available secondary hosts are commonly weed species (Narayanasamy, 2011), which may serve as important sources of inoculum for agricultural crops that are sown or planted in the following season. Outbreaks of crop diseases caused by fungi are typically monitored based on observations of disease symptoms or fungal spore counts, making early preventative control measures (i.e., fungicide application) difficult or impossible to implement (Lindahl and Kuske, 2013). Epidemiological studies have highlighted the need for eradication of potential sources of inoculum (i.e., weeds) between growing seasons so as to restrict the incidence and spread of disease to newly planted crops (Narayanasamy, 2011). These studies have focused on a small number of pathogen species infecting major crops; however, little is known of the larger pool of fungal species that co-inhabit the plant phyllosphere (leaf surface and interior). Pathogens constitute a relatively minor proportion of a fungal community—or mycobiome (Sapkota et al., 2015; Donovan et al., 2018). Limited information on the composition of plant mycobiomes is available, with the majority of mycobiome surveys focusing on soil and human body environments (Donovan et al., 2018). Plant pathogens are not commonly featured, despite occasional reports in non-plant mycobiomes (Weyrich et al., 2017; Donovan et al., 2018). A study by Sapkota et al. (2015) found most fungal species to be common to all host cereal crops, with the exception of a few crop-specific pathogens, indicating that the majority of fungi in leaves are not host-specific and that host-specific pathogens live in a “sea” of non-specific fungi. How plant-associated mycobiomes react to inter-species interactions, environmental conditions, host genotype, and agronomic practices is largely unexplored.

Over 99,000 fungal species are documented; however, total diversity is estimated to be between 2.2 and 3.8 million species (Hawksworth and Lücking, 2017). Traditional culture-dependent studies have revealed an immense diversity of fungal communities colonizing the plant phyllosphere, although more recent metagenomic studies indicate that this may be underestimated (Peršoh, 2015). Phyllosphere mycobiomes are highly variable among leaves within an individual plant, with factors such as leaf position, canopy height, and leaf age shown to influence leaf-to-leaf variability (Kinkel, 1997). An overlap between fungi found in the phyllosphere and the air spora has also been reported (Levetin and Dorsey, 2006). A study on beech tree (*Fagus sylvatica*) looking at variability at four different spatial scales (tree, branch, group of leaves, and individual leaf) found the majority of variation occurred at the smallest spatial scale (i.e., between individual leaves), with intra-host variability of phyllosphere fungal populations distinctly greater than inter-host variability (Cordier et al., 2012). However, within a single tree canopy, mycobiome profiles become more similar with decreasing distance, suggesting that these differences may be minimized for a single sapling when it is small and has only a few leaves. When analyzing differences between individual trees, dissimilarity between mycobiomes was linked with genetic rather than geographic distance between trees (Cordier et al., 2012). Several studies have also found that genetic makeup of the plant host at both the species and cultivar/ecotype level was the major factor influencing fungal diversity on plant leaves (Joshee et al., 2009; Bálint et al., 2013; Hunter et al., 2015; Sapkota et al., 2015), with spatial and seasonal factors also having significant but lesser impacts. While Sapkota et al. (2015) found crop genotype was the principal factor in explaining mycobiome diversity in cereal phyllospheres, within each of the individual crops studied (wheat, winter, and spring barley), location also played an important role. Blixt et al. (2010) found that variation of community composition was greater between fields of diseased wheat (*Triticum aestivum*) than within fields as did Zimmerman and Vitousek (2012) who found that among-site diversity of a single tree species (*Metrosideros polymorpha*) contributed more than within-site diversity to the overall fungal community richness. In addition to spatial, temporal, and genetic factors, phyllosphere mycobiomes are also influenced by management factors, with fungicide use shown to have an impact on the composition of cereal leaf mycobiomes (Karlsson et al., 2014; Sapkota et al., 2015).

In this study, we identified the plant pathogens present in the phyllosphere mycobiomes of commonly found weed species adjacent to cropping fields. We also report differences in weed mycobiome composition in response to host species and spatial/climatic factors. This information on weed host-specific or region-specific association with pathogen species may become an increasingly important factor in developing new methods for crop disease management in the future.

## Materials and Methods

### Field Sampling

**FIGURE 1:**
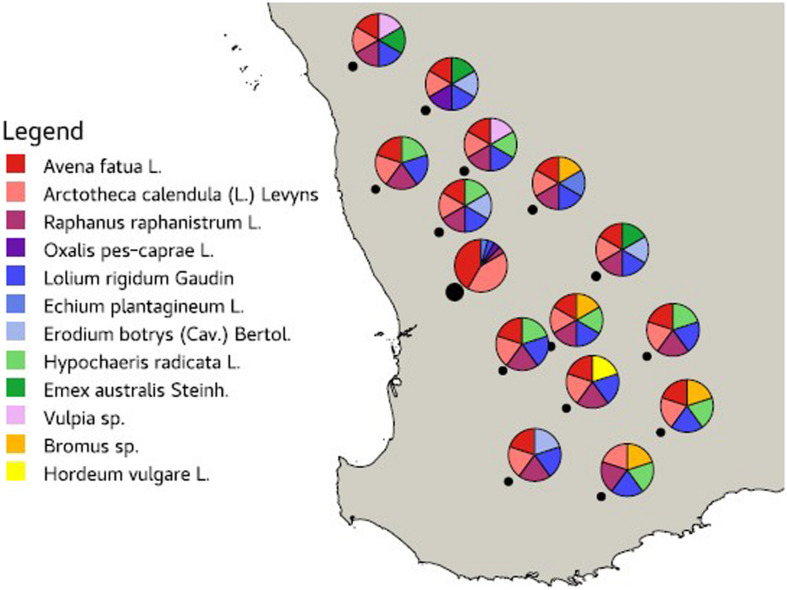
Map of the Western Australian grain belt showing the 15 sampling locations (black dot) and the weed species sampled at each site.

Leaves from 12 common agricultural weed species (Table 1) were sampled from 15 locations across the Western Australian grain belt (Figure 1) over a 5-day period during autumn, 2016. All sites were located on road verges adjacent to cropping paddocks and selected for maximum number of weed species present. Environmental and spatial data were recorded for each site (Table 2), with climatic data from 1950 to 2020. The optimal number of individual plants required per site to be representative of the fungal biota present needed to be determined. Therefore, 10 leaves of two weed species (*Lolium rigidum* and *Raphanus raphanistrum*) were sampled from one site (Yoting) and the preliminary metagenome sequencing results were used to benchmark the appropriate number of leaves for sequencing (Supplementary Figure 1). Thus, for each weed species sampled at each site, one uppermost leaf from 10 individual plants were stored at 4°C during transport to the lab (24–48 h), then at −20°C. Each set of 10 leaves was randomly sub-sampled using a 5-mm disc punch and combined in a 5-ml sample vial until full prior to DNA sequencing.

**TABLE 1:** Sampling incidence of 12 common weed species from 15 locations within the Western Australian grain belt.

Species	Family	Group	Common name	No. locations sampled
*Arctotheca calendula* L.	Asteraceae	Dicotyledon	Capeweed	14
*Avena fatua* L.	Poaceae	Monocotyledon	Wild oats	13
*Bromus* spp.	Poaceae	Monocotyledon	Brome grass	4
*Echium plantagineum* L.	Boraginaceae	Dicotyledon	Patterson’s curse	2
*Emex australis* Steinh.	Polygonaceae	Dicotyledon	Doublegee	3
*Erodium* spp.	Geraniaceae	Dicotyledon	Erodium	4
*Hordeum* spp.	Poaceae	Monocotyledon	Barley grass	1
*Hypochaeris radicata* L.	Asteraceae	Dicotyledon	Flatweed	8
*Lolium rigidum* Gaud	Poaceae	Monocotyledon	Annual ryegrass	14
*Oxalis pes-caprae* L.	Oxalidaceae	Dicotyledon	Soursob	1
*Raphanus raphanistrum* L.	Brassicaceae	Dicotyledon	Wild radish	12
*Vulpia* spp.	Poaceae	Monocotyledon	Silvergrass	2

**TABLE 2:** Spatial and climatic details (averaged over 1950–2020) for each location.

Location	Latitude (°S)	Longitude (°E)	Annual rainfall (mm)	Annual mean temp (°C)	Annual min temp (°C)	Annual max temp (°C)	No. weeds sampled
Badgingarra	−30.2111	115.4599	521	19.2	12.3	25.6	5
Borden	−34.0256	118.2634	377	15.8	9.8	21.7	5
Buntine	−29.9852	116.5618	319	19.4	12.2	26.5	6
Harrismith	−32.9292	117.8300	356	16.5	9.6	22.8	5
Hyden	−32.2848	118.8303	342	17.1	9.9	24.5	5
Kalannie	−30.4651	117.4111	307	19.2	12.0	26.0	6
Kojonup	−33.8383	117.1103	505	15.5	9.1	21.6	5
Morawa	−29.0817	115.9865	308	20.0	12.8	27.6	6
Mullewa	−28.6862	115.1780	352	19.6	13.0	26.6	6
Newdegate	−33.2284	119.0020	357	16.0	9.5	22.9	5
Nungarin	−31.2907	118.1990	296	18.2	11.4	25.3	6
Pingelly	−32.4618	117.0406	427	16.7	9.9	23.7	5
Toodyay	−31.4870	116.4431	485	18.1	11.2	24.9	3
Walebing	−30.7444	116.2491	432	18.3	11.6	25.1	6
Yoting	−32.1621	117.6333	325	17.4	10.2	24.2	4

*Data sourced from https://www.longpaddock.qld.gov.au/silo/.*

### DNA Extraction and Quantification

DNA was extracted using a QIAmp Plant Mini Kit (Qiagen, Venlo) with a modified protocol including (1) 600 μl of API digestion buffer; (2) overnight sample digestion at 65°C; and (3) the following day, addition of 195 μl of P3 to digests prior to putting on ice. A total volume of 400 μl was placed in a QIAcube, after which the remaining QIAmp Plant Mini Kit protocol was followed. Genomic DNA (gDNA) extracts were eluted in 100 μl of AE buffer and stored at −20°C, and then quantified and assessed for quality via real-time quantitative polymerase chain reaction (qPCR) at three dilutions (1:1, 1:10, and 1:100). Primer pairs were designed to amplify a highly conserved region of the 26S gene between the D1/D2 domains (Q1_F 5′ GTTGTTTGGGAATGCAGCTC 3′ and QB3_R 5′ AGTGCTTTTCATCTTTCCCTCAC 3′). qPCR was performed in 25-μl reactions containing 1 × PCR Gold Buffer, 2.5 mM MgCl_2_, 0.4 mg/ml BSA, 0.25 mM of each dNTP, 0.4 μM of forward and reverse primer, 0.25 μl of AmpliTaq Gold, 0.6 μl of SYBR Green, and 2 μl of gDNA. The qPCR cycling conditions included an initial heat denaturation at 95°C for 5 min, 40 cycles of 95°C for 30 s, 52°C for 30 s, and 72°C for 45 s, and then a final extension at 72°C for 10 min. From the qPCR results, an optimal DNA concentration was selected for DNA sequencing, which was free of inhibition and yielded DNA of sufficient quality, as reported to facilitate reproducible quantitative data by (Murray et al., 2011).

### High-Throughput DNA Sequencing

The D2 and ITS2 amplicons were sequenced on an Illumina MiSeq system utilizing previously published primers that were modified with a unique 8-bp Multiplex Identifier tag (MID-tag) and MiSeq adaptors for paired-end sequencing. For the D2 domain primers, U1_F (Putignani et al., 2008) and NL4_R (Kurtzman and Robnett, 1998) were utilized, and for the ITS2 region, fITS7_F (Ihrmark et al., 2012) and ITS4_R (White et al., 1990) were used. Independent MID-tagged qPCR setup for samples and controls were prepared in a physically separate ultra-clean laboratory and were carried out using each primer set in 25-μl reactions containing 1 × PCR Gold Buffer, 2.5 mM MgCl_2_, 0.4 mg/ml BSA, 0.25 mM of each dNTP, 0.4 μM of forward and reverse MID-tag primer, 0.25 μl of AmpliTaq Gold, 0.6 μl of SYBR Green, and 2 μl of gDNA. The cycling conditions for qPCR using the U1_F/NL4_R (52°C annealing) and fITS7_F/ITS4_R (54°C annealing) primer sets were as follows: initial heat denaturation at 95°C for 5 min, followed by 40 cycles of 95°C for 30 s; 52°C or 54°C for 30 s (annealing step); and 72°C for 45 s followed by final extension at 72°C for 10 min. Multiplex Identifier-tagged PCR amplicons were generated in duplicate for each sample and pooled together to minimize the effects of PCR stochasticity. The pooled amplicons were quantified on a LabChip (with high-sensitivity chip) and then combined to produce a final library of equimolar ratio per sample. The final library was then purified on a PippinPrep with a size selection gate of 300–600 base pair capture following the manufacturer’s protocol (PerkinElmer). The purified library was diluted with purified water and re-run on the LabChip to determine the volume required (2 nM) for Illumina MiSeq paired-end sequencing. For each MID-tagged qPCR assay, extraction and PCR controls were included, and if found to contain amplifiable DNA, these reactions were incorporated into the pooled MID-tagged DNA sequencing library. Illumina MiSeq sequencing was performed using a MiSeq Reagent Kit v2 (500 cycles) 250-bp paired-end protocol as per the manufacturer’s instruction. Paired-end reads were stitched using Illumina’s MiSeq Reporter software.

### DNA Sequence Quality Filtering and Analyses

Stitched sequences were separated from unstitched, which had low overall phred scores and were discarded. MID-tags and gene-specific primers were trimmed from the sequences allowing for no mismatch in length or base composition using QIIME1 (extract_barcodes.py) and de-multiplexed using QIIME2 (Caporaso et al., 2010). De-multiplexed reads were then trimmed and filtered using v1.9 (≤6 undetermined bases, quality cutoffs: 5′ = 25 and 3′ = 22) (Martin, 2011). Reads were then error-corrected using deblur (Amir et al., 2017) within QIIME2 (Caporaso et al., 2010), using a custom database of ITS [combining UNITE (Kõljalg et al., 2013), ITS2 (Ankenbrand et al., 2015), and NCBI bioproject: PRJNA177353] and LSU [combining RDP (Cole et al., 2013) and NCBI bioproject: PRJNA51803] sequences, but discarding those below 258 bp for ITS and 240 bp for D2 sequences. Similar sequences to these clustered consensus sequences in the NCBI nt and GSS databases were also found using BLAST + v2.2.6 (blastn). The BLAST databases were filtered before searching to exclude sequences with NCBI taxonomic IDs in the subtree below “12908” (unknown and environmental samples) or containing the keyword “Uncultured” in the sequence name.

### Data Analysis

BLAST outputs were analyzed using MEGAN v6.11.2 (Huson et al., 2016) (Weighted LCA, Min Score: 50, Max Expected: 1e-5, Min Percent Identity: 70, Top Percent: 10.0, Min Support Percent: 0.05, Min Support: 1, Percent to cover: 50). Taxonomic lineages for OTUs were found and manipulated using custom scripts^1^ based on the NCBI taxonomy database. OTUs with taxonomic assignments were associated with the samples and summarized to generate presence–absence profiles. Presence–absence profiles of fungal taxa were generated for each sample using a cumulative approach, whereby the presence of a low-level taxon also automatically assigned presence to its higher-level taxa. Patterns of variance were investigated using principal components analysis (PCA) using the scikit-learn python package (Pedregosa et al., 2011) as well as by performing canonical correspondence analysis (CCA) using the R package “vegan” (Oksanen et al., 2019). Species richness was also measured using the Shannon–Weaver and Simpson diversity indices in the R package “vegan.” To infer the specific identities of OTUs, OTU sequences were searched with BLAST against the custom database developed for this study (see above). BLAST matches were filtered to contain alignments with greater than 95% identical matches, and covering at least 90% of the OTU sequence.

## Results and Discussion

### Crop-Zone Weeds Are Host to a Wide Range of Plant-Associated Fungi

**FIGURE 2:**
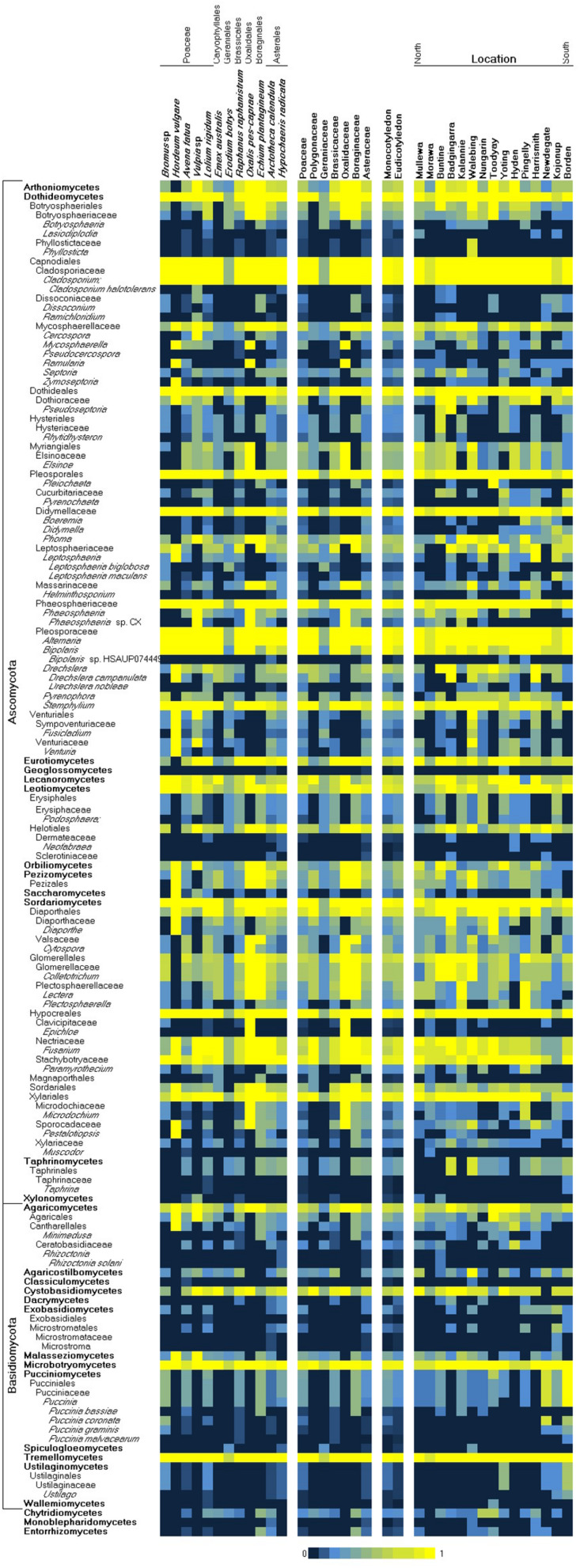
Heatmap of the observed presence of common fungal pathogen genera/species by weed host species and collection location (left/North to right/South). A presence score from 0 to 1 (shown in blue–yellow) indicates the proportion of observed presence of a taxon across all samples of a common species or location, where 0 indicates total absence and 1 indicates ubiquity.

Overall, 2391 and 1819 OTUs were detected from the sequenced ITS and D2 loci, respectively (Supplementary Table 1). As an indicator of the specificity of identification, 1231 ITS and 1086 D2 OTUs were directly assigned to NCBI taxonomy identifiers at genus level or lower via BLASTN to Genbank (nt and GSS), while 7 and 15 could not be assigned to any taxonomic identifiers (Supplementary Table 2). Most OTUs were assigned to taxa at or below the fungal classes Dothideomycetes, Tremellomycetes, and Sordariomycetes (Supplementary Table 3). Species richness was highest in weeds within the Brassicaceae and Polygonacae, followed by other host families that were not dissimilar. Among the fungal species taxa detected in weed samples, the heteroecious rusts of the *Puccinia* spp. (Zhao et al., 2016) (*Puccinia graminis*, *Puccinia bassiae*, *Puccinia coronata*, and *Puccinia malvacearum*) were prominently represented across multiple West Australian weed hosts (Supplementary Table 3). These reports of common Australian weeds as alternate hosts for the *Puccinia* rusts are analogous to a growing collection of reports across Europe, the United States, and Asia (Roelfs, 1982; Jin et al., 2010; Wang and Chen, 2013; Zhao et al., 2016). In this study, *P. graminis* was observed on *Bromus*; *P. bassiae* on *Echium* and *Avena*; *P. coronata* on *Bromus* and *Lolium*; and *P. malvacearum* on *Bromus*, *Lolium*, *Avena*, *Echium*, and *Raphanus*. Additional species of agricultural relevance that were identified tended to infect multiple hosts, including the following: *Leptosphaeria maculans*, *Leptosphaeria biglobosa*, *Drechslera campanulata* (syn. *Pyrenophora semeniperda*; ring spot of oat), and *Drechslera nobleae* (syn. *Pyrenophora phaeocomes*, pathogen of rye and pastures). Species endemic to Australia, including several species associated with native eucalypts (*Cladoriella eucalypti, Fusculina eucalypti, Plectosphaera eucalypti, Phacidiella eucalypti, Phaeococcomyces eucalypti*, and *Saccharata eucalyptorum*) (Supplementary Table 2), were detected, which were consistent with the local environment but unrelated to crop production. Several genera that contain well-known crop pathogen species were also detected, however, their species-level identities were inconclusive. These included *Cladosporium*, *Zymoseptoria*, *Phaeosphaeria*, *Bipolaris*, *Pyrenophora*, *Venturia*, *Colletotrichum*, *Fusarium*, *Taphrina*, and *Ustilago*. We looked for (using covariance analysis of presence–absence variation) consistent association of two or more species across samples, as well as the inverse mutual exclusion of species (Supplementary Table 4), but unlike similar previous studies (Sapkota et al., 2017), we did not observe strong evidence of species complexes or potential bio-control agents across these samples. While culture-independent methods, which are based on the extraction and analysis of nucleic acids, can provide a comprehensive overview of microbial communities, they have drawbacks such as the detection of DNA from dead cells (Müller and Ruppel, 2014) and dependence on well-annotated databases (Blackwell, 2011; Aylward et al., 2017). It has been reported that up to 20% of fungal sequences in major databases such as GenBank may be misidentified (Bridge et al., 2003; Nilsson et al., 2008). However, the internal transcribed spacer regions (ITS1 and ITS2) have been demonstrated to be taxonomic markers for fungi due to their length and discriminative sequence variation (Lindahl and Kuske, 2013). Nevertheless, an issue we encountered with the taxonomic mapping of OTUs was that mappings to the species level were not consistently achieved. Species level (or lower taxa) would be required in order to reliably assign a crop disease to an OTU and infer its potential host range. Species-level mapping was dependent on multiple factors including representation bias across different taxa in sequence databases, variable map-ability of reads across different taxa, and potential for some species to be present but undetectable. In this study, OTU alignments more often supported the genus-level over species-level mappings; hence, this limited our ability to infer fully the disease risks posed by crop-zone weeds. We have presented a taxonomic summary of genera likely to contain plant-pathogenic species in Figure 2, although in most cases, the presence of pathogenic species was inconclusive, as endophytic species may have also been detected.

### Regional Biases

**FIGURE 3:**
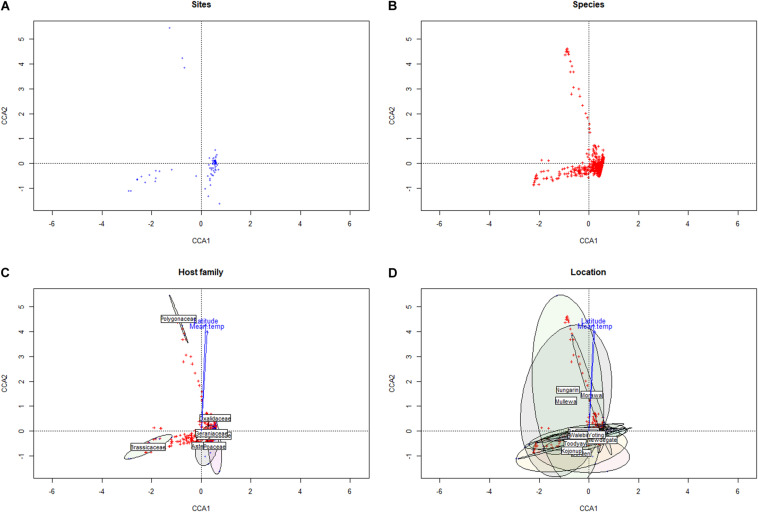
Canonical correspondence analysis of sampled sites **(A)** and microbial taxa **(B)** versus climate conditions and geographic locations, with groupings presented by taxonomic family **(C)** and by location **(D)**.

Within the Western Australian regions sampled, there appeared to be an overall trend for a relatively increased presence of Ascomycota in northern sample sites and a corresponding relative increase in Basidiomycota (particularly biotrophic rusts of the *Puccinia* spp.) in southern sites (Figure 2). Multivariate clustering of samples tended to form groups based on geographic locations rather than by the weed host (Supplementary File 1), suggesting that location and/or climate zone was a major factor influencing the weed phyllosphere. This was also supported by CCA (Figure 3 and Supplementary File 2), which indicated that the phyllosphere composition was influenced by the continuous variables, latitude and mean temperature. Species distributions in the CCA corroborated the initial observation of a necrotrophic bias in hotter and dryer northern regions and a corresponding biotrophic bias in cooler and wetter southern regions (Figure 3 and Supplementary File 2). CCA also indicated clustering of hosts of the Polygonaceae family with a strong association with northern latitudes and increased temperatures, which was due to the three *Emex* (syn. doublegee) samples having been exclusively obtained from sites in the northern wheatbelt (Mullewa, Morawa, and Nungarin). However, a host cluster by Brassicaceae (composed of 13 *R. raphanistrum* samples) along CCA1 could not be explained by latitude or temperature vectors and suggests that unique host-specific pathogens may be present on this weed species. The other host families were represented at all sites and did not exhibit any clustering by CCA.

This approximate north-to-south division may reflect that the northern regions in this study are warmer and drier, which may suppress saprophytic and/or pathogen-suppressive microbial activity that would break down the stubble containing pathogen inoculum—a common problem with cereal necrotrophs (typically of the Ascomycota). Conversely, the southernmost regions of WA are cooler and have increased rainfall, which likely increases the spread and survivability of heteromecic/obligate biotrophic pathogens (typically of the Basidiomycota). Although crop pathogen species were in the relative minority within the phyllosphere, this appears to be consistent to other studies linking climate zones to the distribution of fungal pathogens (Bebber et al., 2014; Fisher et al., 2018).

## Conclusion

This survey of the weed phyllospheres local to cereal crop-growing regions of Western Australia demonstrates the utility of this approach for the monitoring of plant pathogenic species and could be adapted for the purpose of monitoring for pathogen reservoirs and emerging crop disease risks. We report the presence of several important crop pathogen species within the phyllospheres of local weed hosts and observe that host range and climate zone are both important factors in determining their geographic distributions.

## Data Availability Statement

Sequence data was deposited in NCBI BioProject: https://www.ncbi.nlm.nih.gov/bioproject/672330.

## Author Contributions

PM, JH, and MG contributed to the conception and design of the study. PM conducted the field sampling. NW contributed to DNA extraction and sequencing. DJ and JH contributed to DNA sequence quality filtering and data analysis. PM, JH, DJ, and NW contributed to the manuscript. All authors contributed to the article and approved the submitted version.

## Conflict of Interest

The authors declare that the research was conducted in the absence of any commercial or financial relationships that could be construed as a potential conflict of interest.

## Footnotes

1 https://github.com/darcyabjones/acc-to-tax

## Supplementary Material

Supplementary Figure 1 Fungal species accumulation curves across 10 samples of *Lolium rigidum* and *Raphanus raphanistrum* leaves sampled from Yoting.

Supplementary Table 1 OTU frequencies for ITS and D2 regions versus host taxonomy and sample locations.

Supplementary Table 2 Assignment of taxa to OTUs via BLAST.

Supplementary Table 3 Combined ITS and D2 relative frequencies of microbial taxa versus host taxonomy and sample locations. Each microbial taxon detected by either an ITS or D2 OTU sequence is expressed in terms of a number ranging from 0 to 1, where 1 indicates universal occurrence across host taxa or sample locations, and 0 indicates no occurrences.

Supplementary Table 4 Co-variance tests for association of taxon pairs across samples. Taxon pairs with high or low co-variance may indicate biologically relevant associations or mutually exclusions across samples in this study.

Supplementary File 1 Multivariate analyses of microbial taxa testing for relationships versus host species or sample locations.

Supplementary File 2 Canonical correspondence analyses of microbial taxa testing for relationships between host taxa, pathogen taxa, sample location and climate.
