# Supplementary figures and images for: Crop-Zone Weed Mycobiomes of the South-Western Australian Grain Belt
Source: Front Microbiol. 2020 Nov 24;11:581592. doi: 10.3389/fmicb.2020.581592 (PMC7721668; doi:10.3389/fmicb.2020.581592)

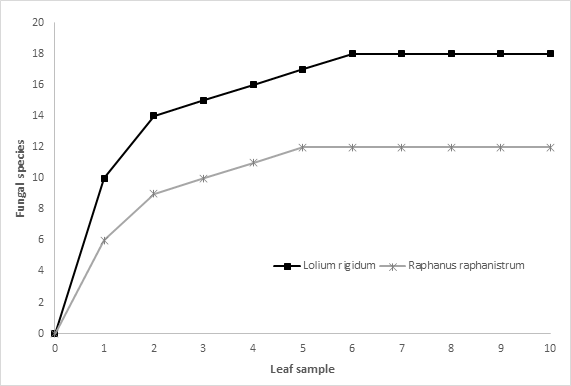

Supplement: Supplementary Figure 1 — Fungal species accumulation curves across 10 samples of Lolium rigidum and Raphanus raphanistrum leaves sampled from Yoting. [file Image_1.PNG]

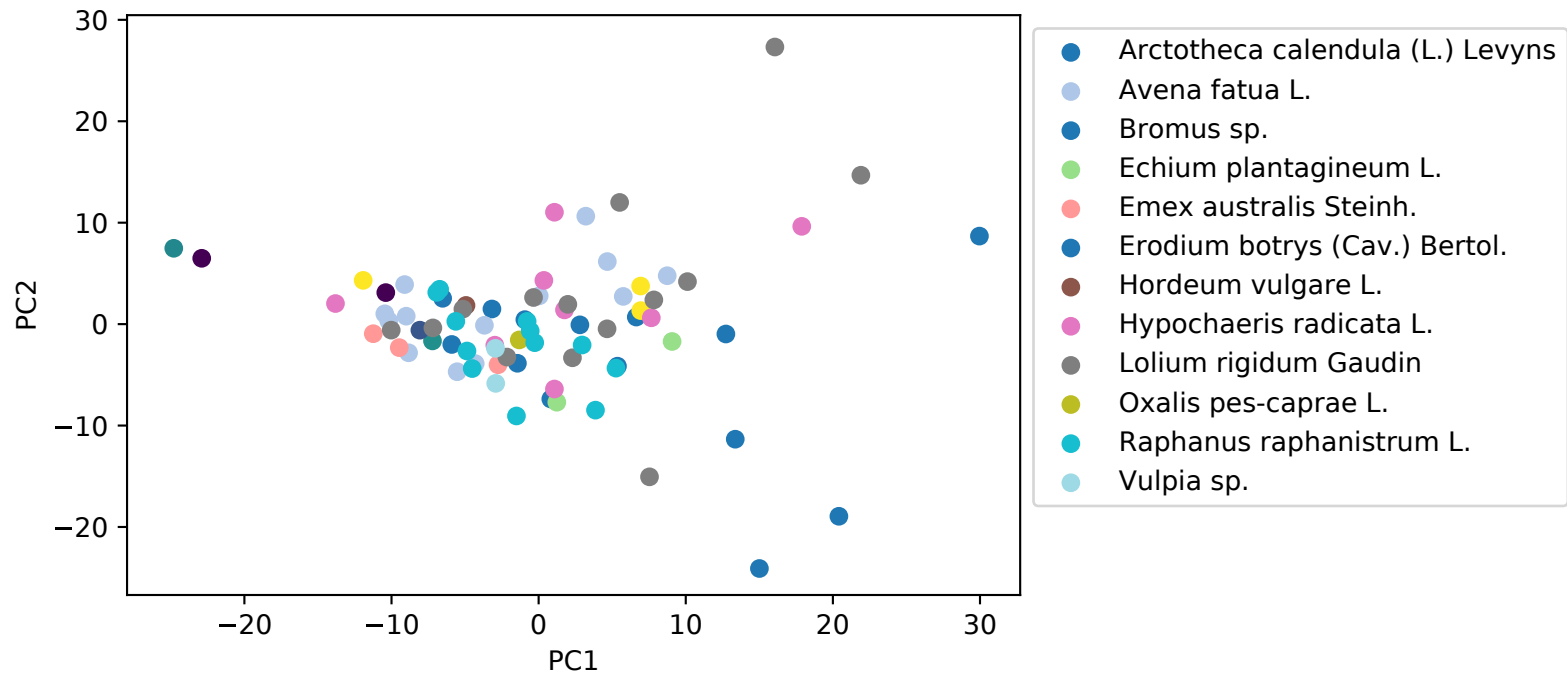

Supplement: Supplementary File 2 — Canonical correspondence analyses of microbial taxa testing for relationships between host taxa, pathogen taxa, sample location and climate. [file Data_Sheet_1.ZIP › Supplmentary File 5 Multivariate analyses/By host - PC1 vs PC2.pdf]

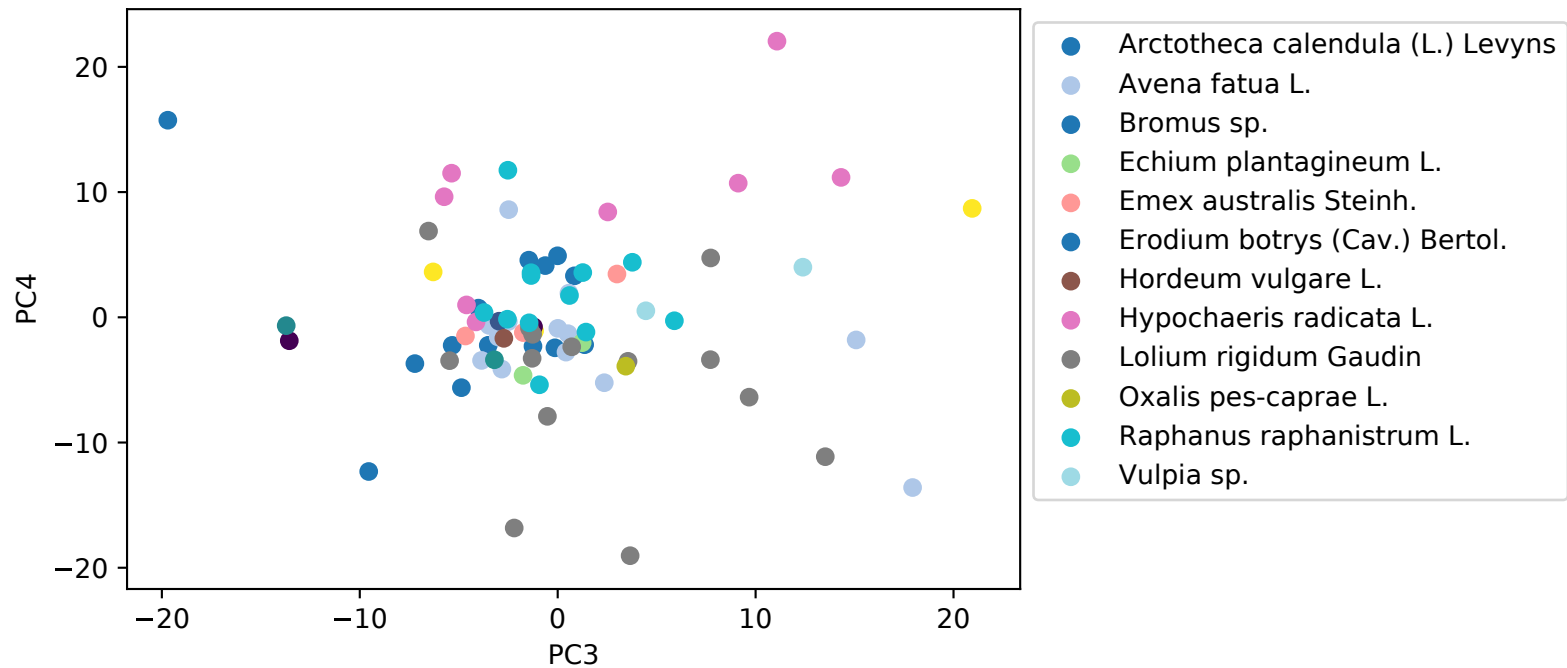

Supplement: Supplementary File 2 — Canonical correspondence analyses of microbial taxa testing for relationships between host taxa, pathogen taxa, sample location and climate. [file Data_Sheet_1.ZIP › Supplmentary File 5 Multivariate analyses/By host - PC3 vs PC4.pdf]

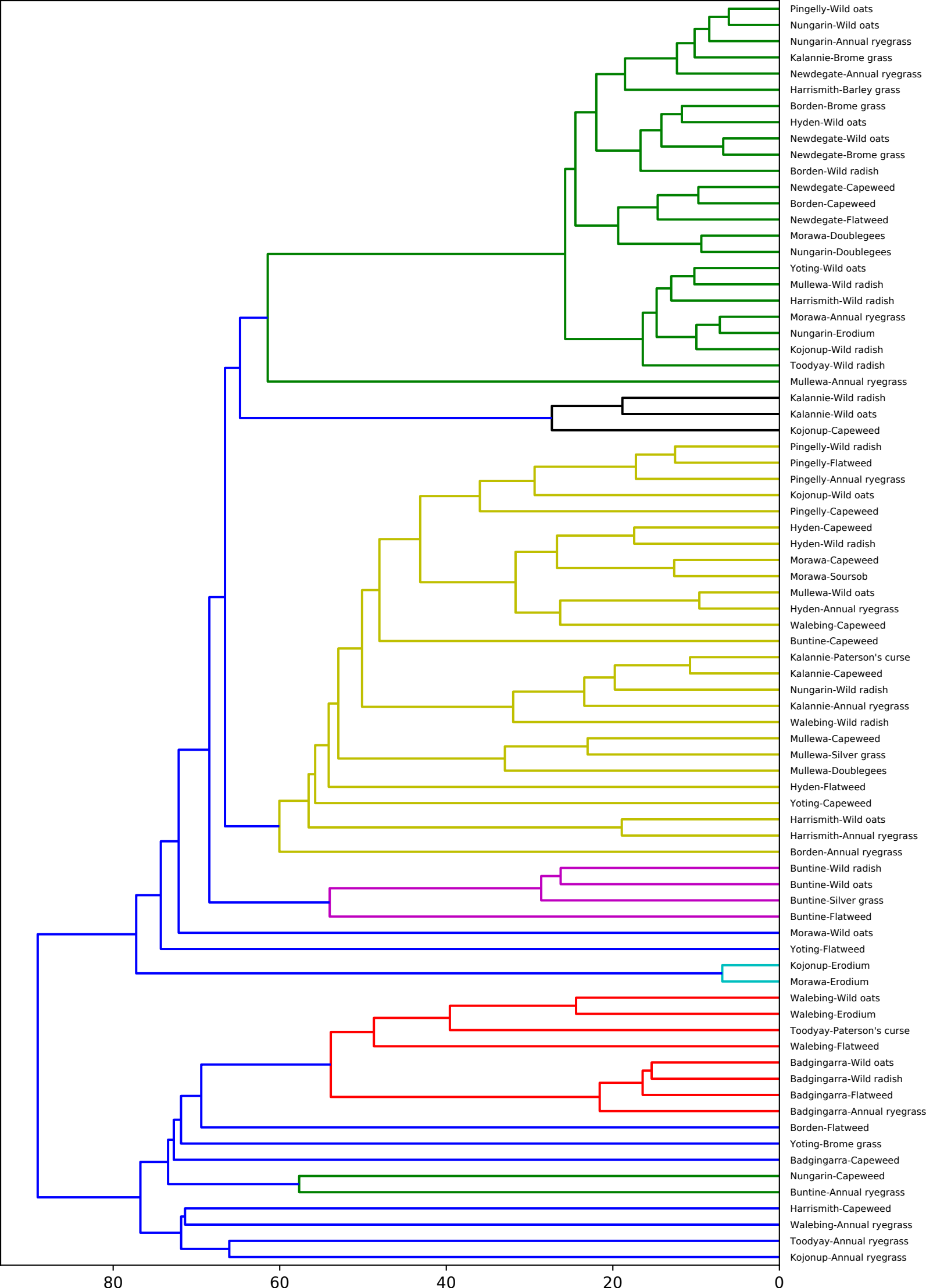

Supplement: Supplementary File 2 — Canonical correspondence analyses of microbial taxa testing for relationships between host taxa, pathogen taxa, sample location and climate. [file Data_Sheet_1.ZIP › Supplmentary File 5 Multivariate analyses/By location - Dendogram.pdf]

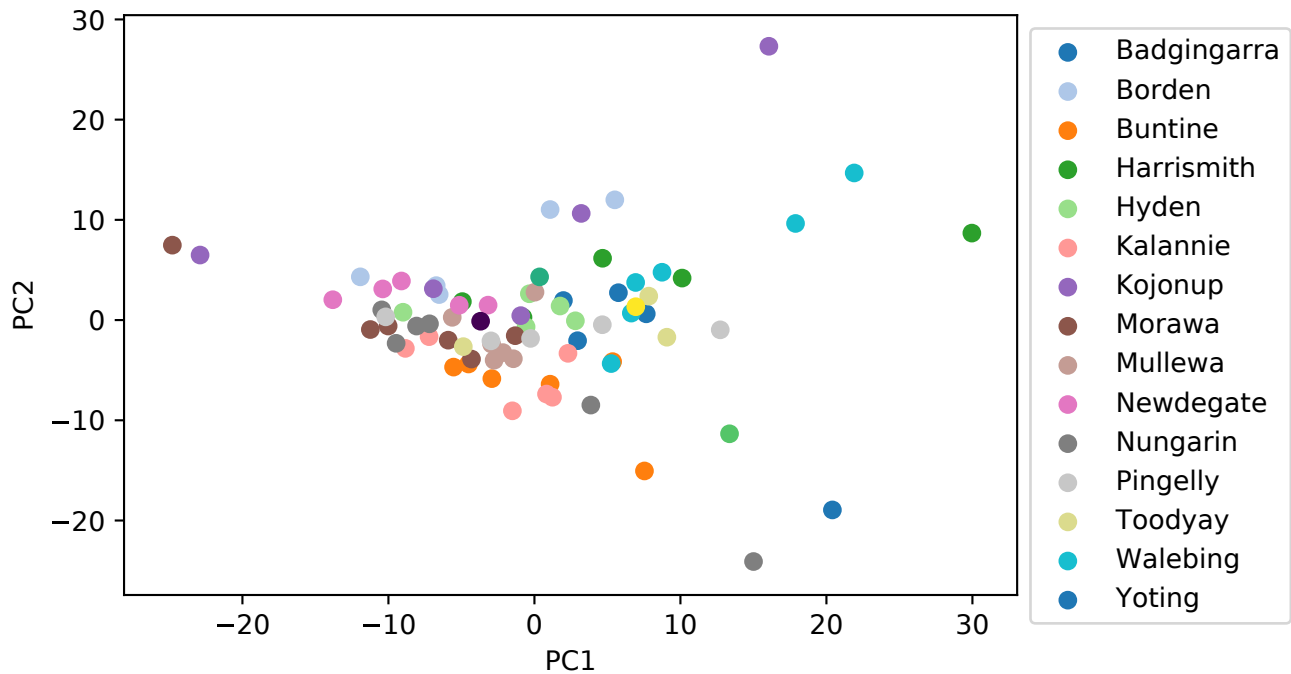

Supplement: Supplementary File 2 — Canonical correspondence analyses of microbial taxa testing for relationships between host taxa, pathogen taxa, sample location and climate. [file Data_Sheet_1.ZIP › Supplmentary File 5 Multivariate analyses/By location - PC1 vs PC2.pdf]

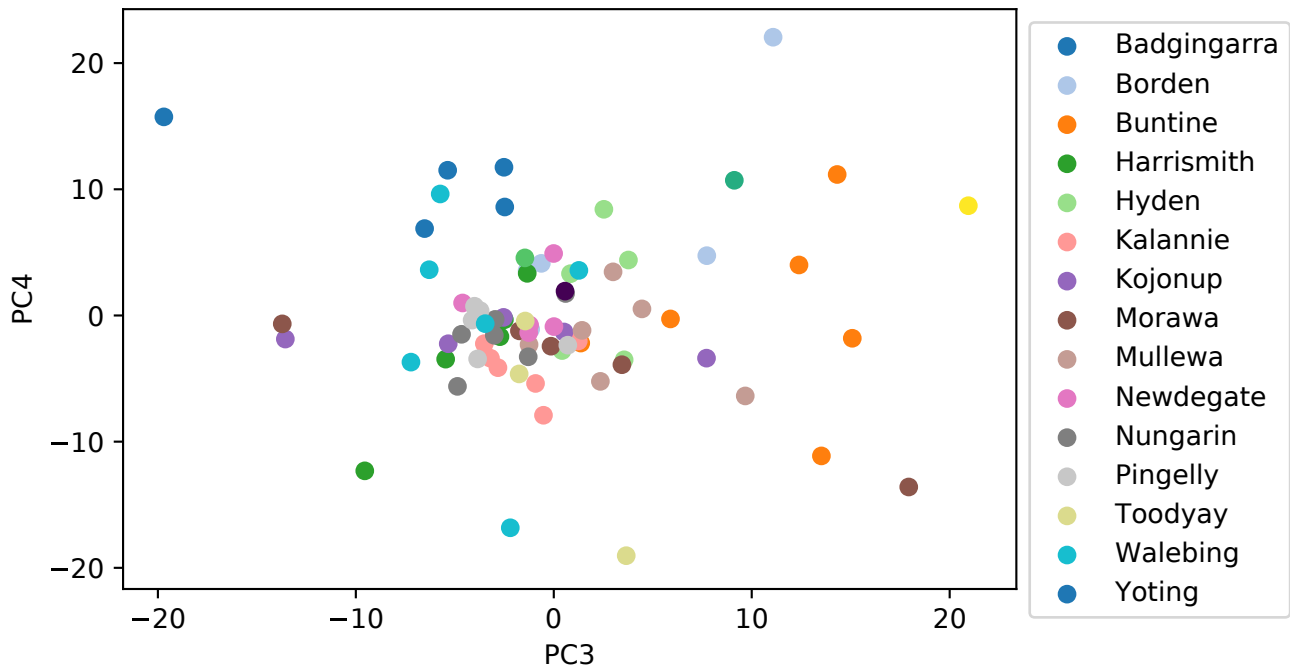

Supplement: Supplementary File 2 — Canonical correspondence analyses of microbial taxa testing for relationships between host taxa, pathogen taxa, sample location and climate. [file Data_Sheet_1.ZIP › Supplmentary File 5 Multivariate analyses/By location - PC3 vs PC4.pdf]
